# Supplementary material for: Light-Activated Room Temperature Surface Acoustic Wave H2S Sensor Based on Bi2S3 Nanoribbons
Source: Sensors (Basel). 2025 Feb 12;25(4):1122. doi: 10.3390/s25041122 (PMC11858874; doi:10.3390/s25041122)
Supplement: Supplementary file 1 [file sensors-25-01122-s001.zip › sensors-3437615-supplementary.pdf]

# *Supplementary Information*

**Light-Activated Room Temperature Surface Acoustic Wave $H_2S$ Sensor Based on $Bi_2S_3$ Nanoribbons**

*Chong Li [1], Sami Ramadan [2], Hao Kan [3,*] and Lina Wang [1,*]*

1 *School of Electronic Engineering, Huainan Normal University, Huainan, 232038, China*
2 *Department of Materials, Imperial College London, London SW7 2AZ, U.K.*
3 *School of Information Science and Engineering, University of Jinan, Jinan, 250022, China*
* *Correspondence: wangln@hnnu.edu.cn*

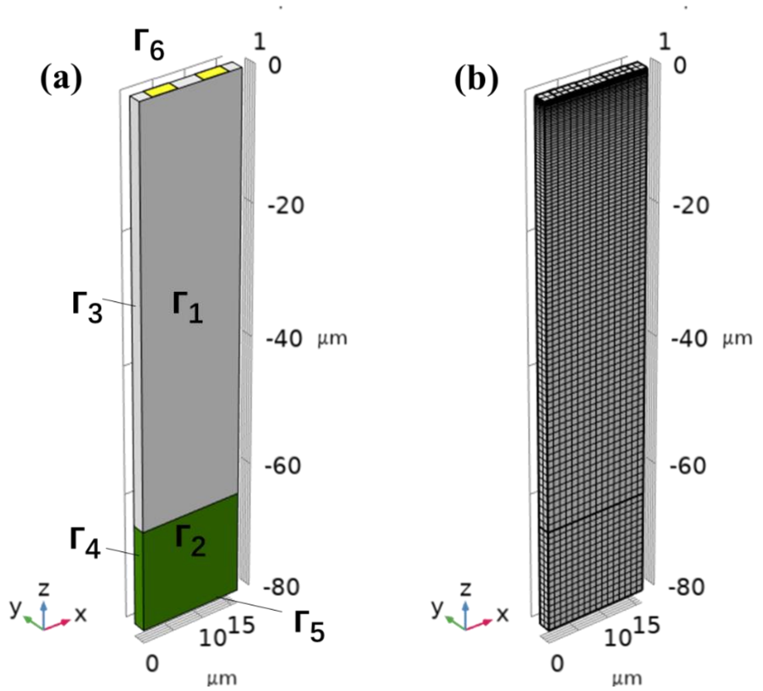


**Figure S1.** (a) Finite element simulation model structure of SAW device; (b) Model meshing.

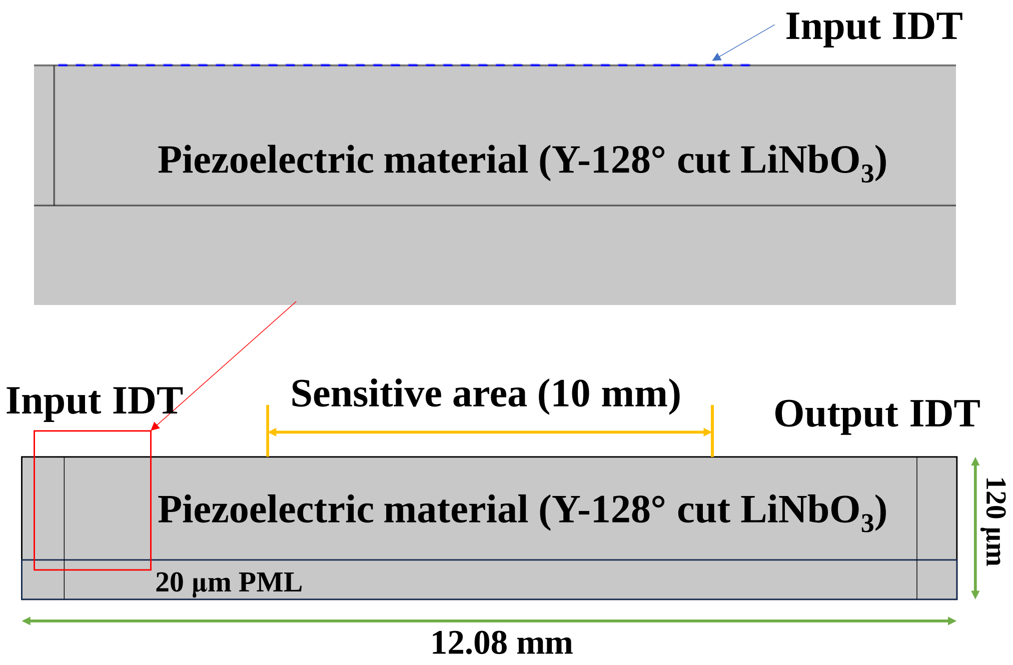


**Figure S2.** 2D computational domain for SAW device.

**Table S1.** Comparison of ligand substitution responses for different salt solutions

| $H_2S$ concentration (ppm) | Untreated response (kHz) | $NaNO_2$ treated response (kHz) | $Zn(NO_3)_2$ treated response (kHz) | $Pb(NO_3)_2$ treated response (kHz) | $Cu(NO_3)_2$ treated response (kHz) |
|---|---|---|---|---|---|
| 2 | -1.179 | -1.732 | -1.594 | -2.91 | -3.066 |
| 5 | -2.115 | -2.193 | -2.857 | -4.01 | -4.31 |
| 10 | -2.992 | -3.45 | -3.877 | -5.5 | -6.085 |
| 20 | -3.763 | -4.138 | -4.841 | -7.6 | -8.252 |
| 30 | -4.354 | -4.762 | -5.317 | -9.281 | -10.58 |
| 40 | -5.016 | -5.266 | -5.873 | -10.242 | -11.772 |
| 50 | -5.588 | -6.26 | -6.323 | -11.298 | -13.548 |

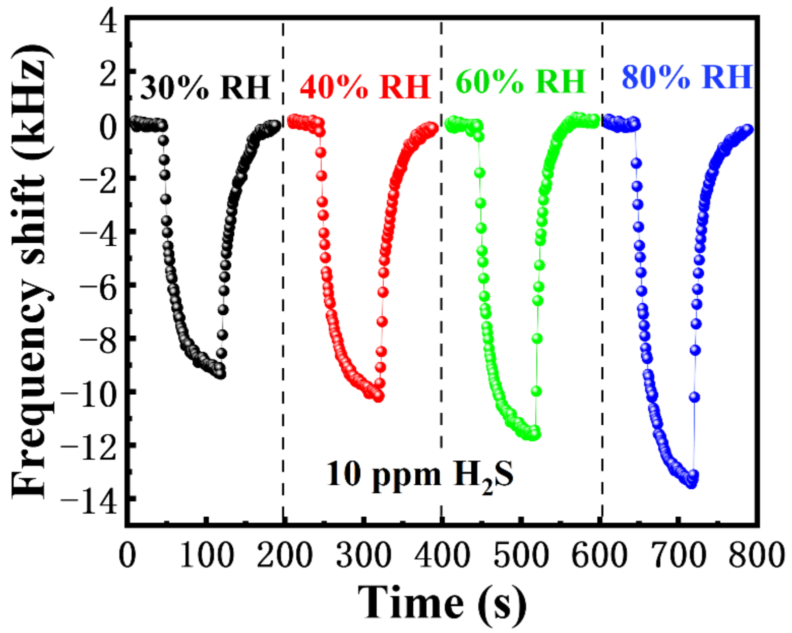


**Figure S3.** Response of the sensor to 10 ppm $H_2S$ at different relative humidity.

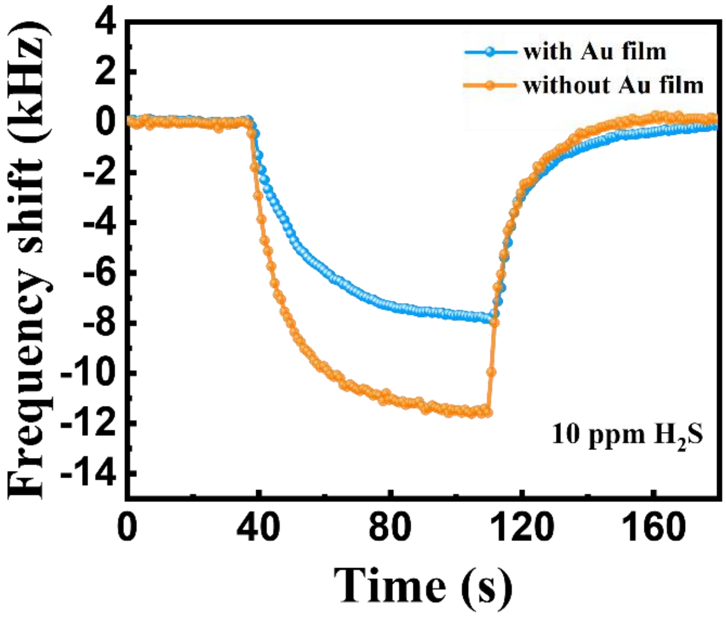


**Figure S4.** The frequency response to 10 ppm of $H_2S$ gas of the sensor without and with Au film.
